# Supplementary material for: Sox5 regulates beta-cell phenotype and is reduced in type 2 diabetes
Source: Nat Commun. 2017 Jun 6;8:15652. doi: 10.1038/ncomms15652 (PMC5467166; doi:10.1038/ncomms15652)
Supplement: Supplementary Information — Supplementary figures, supplementary tables, supplementary methods and supplementary references. [file ncomms15652-s1.pdf]

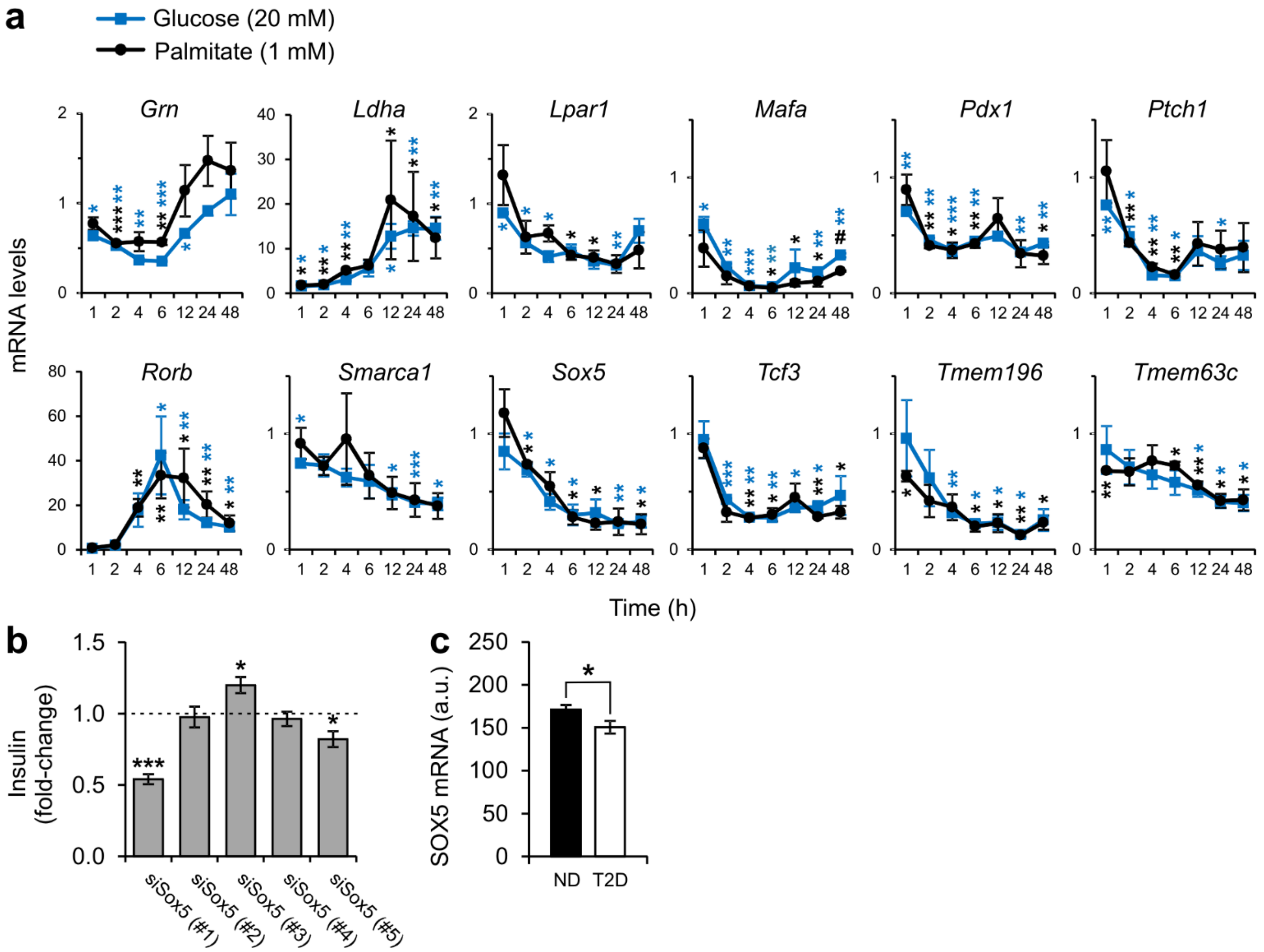

Supplementary Figure 1. Characterization of *Sox5* expression

(a) The expression of putative key regulators of the T2D-associated gene module in rat pancreatic islets during 48 h incubation at 20 mM glucose (blue line) or 1 mM palmitate (black line). Expression at time point 0 was set to 1, and the graphs depict fold-change and statistical significance at time points 1, 2, 6, 8, 12, 24 and 48 h relative to the expression at time point 0 (n=3). #*Mafa* palmitate outlier removed (n=2)

(b) Fold-change of insulin secretion at 16.7 mM glucose 48 h after transfecting INS1-832/13 cells with different siRNAs targeting *Sox5*. Data are compared to cells treated with negative control siRNA (n=4 independent experiments per group). The oligonucleotides were from Life Technologies (#1), Sigma-Aldrich (#2 and #3), and Thermo Scientific (#4 and #5). Knockdown efficiency was for siRNA #1) 72 ± 2%, #2) 62 ± 4%, #3) 68 ± 6%, #4) 58 ± 15% and for #5) 43 ± 9%.

(c) *SOX5* mRNA levels measured by microarray in human islets from non-diabetic (ND; n=82) and T2D (n=41) individuals.

Data are mean ± SEM *p<0.05; **p<0.01; ***p<0.001 using Student's t-test.

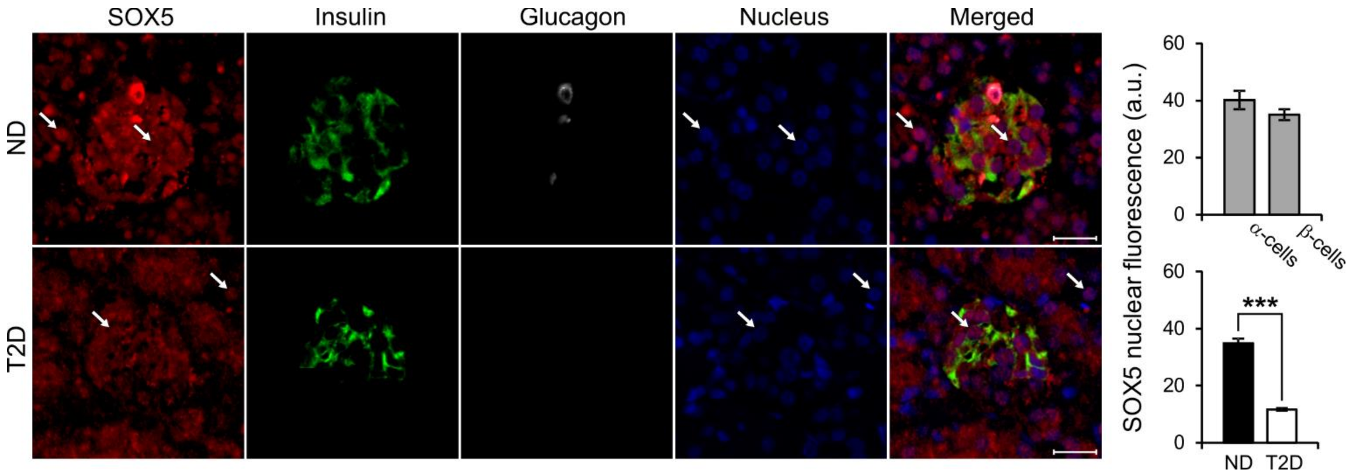

Supplementary Figure 2. Sox5 protein expression in human islets

Representative image of Sox5 protein expression in human pancreas paraffin sections from a non-diabetic and a T2D donor. Examples of nuclear SOX5 staining in β-cells and in exocrine tissue are indicated with white arrows. Scale bar=20 μm. The bar graphs show nuclear SOX5 expression in α-cells (42 cells) and β-cells (56 cells) from a non-diabetic donor and nuclear SOX5 expression in β-cells from a non-diabetic donor (ND; 116 cells) and a T2D donor (113 cells).

Data are mean ± SEM ***$p<0.001$ using Student's t-test.

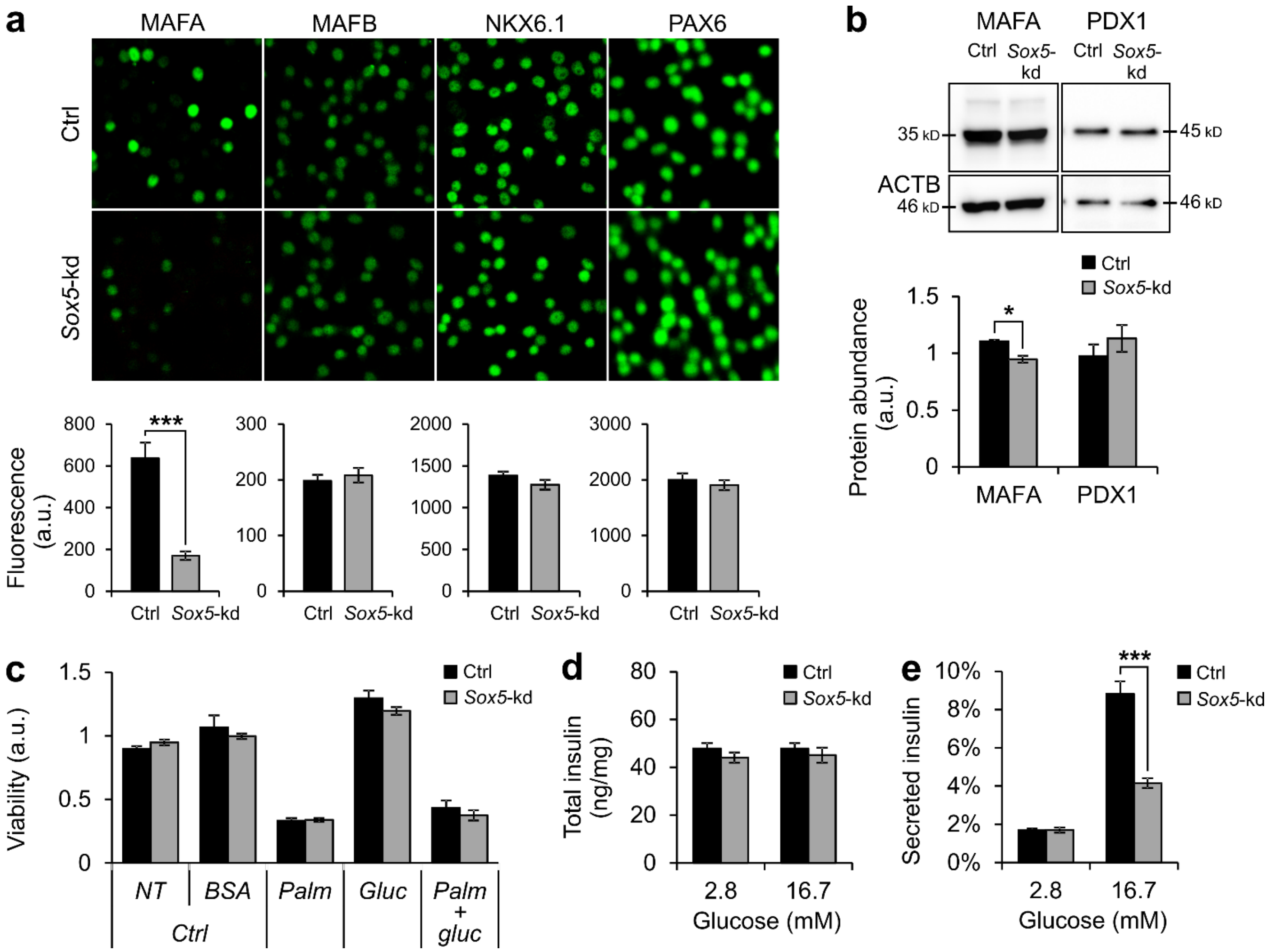

Supplementary Figure 3. Effects of *Sox5* knockdown in INS1-832/13 cells

(a) Immunostaining of *Sox5*-kd and control INS-1 832/13 cells with anti-MafA, anti-MafB, anti-Nkx6.1 and anti-Pax6 antibodies. The bar graphs show the average fluorescence intensity for each antibody (n= 25 cells).

(b) Representative immunoblot of total protein from *Sox5*-kd and control INS-1 832/13 cells. Polyclonal antibodies against MafA and Pdx1 and monoclonal antibody against β-actin (ACTB) were used as indicated. The graph shows average levels of MafA and Pdx1 normalized for β-actin (n=3 for MafA and n=6 for Pdx1).

(c) Cell viability in *Sox5*-kd and ctrl INS-1 832/13 cells treated with 0.5 mM palmitate and/or 20 mM glucose for 48 h (n=3). BSA-treated cells (1% BSA, same concentration as in the palmitate solution) and non-treated (NT) cells were used as controls. Viability was measured with the Aqueous One Solution Cell Proliferation Assay Reagent (Promega).

(d) Total insulin content in INS-1 832/13 cells normalized to total protein content after 1 h incubation at 2.8 or 16.7 mM glucose (n=6).

(e) Secreted insulin normalized to insulin content after 1 h stimulation with 2.8 or 16.7 mM glucose. Data from same samples as in **(f)** (n=6).

Data are mean ± SEM *p<0.05; ***p<0.001 using Student's t-test.

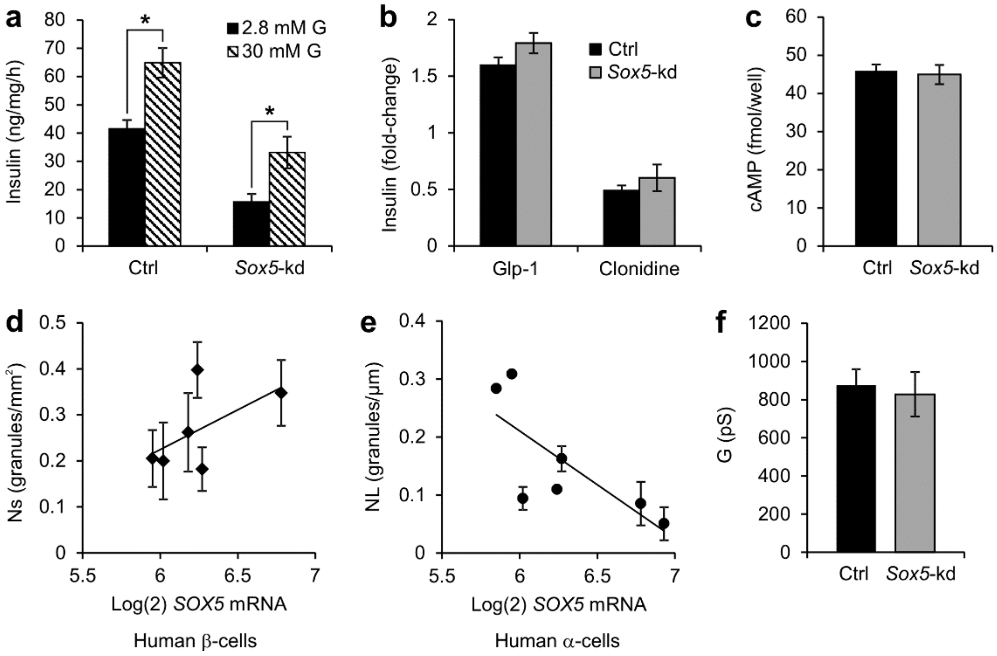

Supplementary Figure 4. Effects of *Sox5* knockdown on insulin secretion pathways and characterization of *SOX5* expression in human islets

The experiments were performed in INS-1 832/13 cells if not stated otherwise. *Sox5*-kd cells were transfected with Sox5 siRNA 48 h before the experiment, and control cells were transfected with negative control siRNA.

(a) The amplifying pathway of insulin secretion was assessed using 250 mM diazoxide to open the K_{ATP} channels and 30 mM K^+ to depolarize the cells at 2.8 or 30 mM glucose. Data show insulin secretion after 1 h incubation at the different conditions in *Sox5*-kd and control cells (n=3).

(b) Insulin secretion in *Sox5*-kd and control cells incubated at 16.7 mM glucose in the presence or absence or 100 nM Glp-1 or 1 mM clonidine as indicated. Data denote fold-change of insulin secretion relative to levels at 16.7 mM glucose (n=3).

(c) Intracellular cAMP levels measured with the cAMP Biotrak Enzyme Immunoassay (EIA) System (GE Healthcare Life Sciences) after 1 h incubation at 16.7 mM glucose (n=3).

(d) Log2-transformed *SOX5* mRNA levels plotted against the number of docked granules as surface density (Ns; granules mm^{-2}) in human ß-cells (n=5-9 cells per donor from 6 donors with T2D). A granule was defined as docked when the center of the granule was located within 150 nm from the plasma membrane.

(e) Log2-transformed *SOX5* mRNA levels plotted against the number of docked glucagon granules measured as Nl (granules μm^{-1}) in human α-cells (n=1-13 cells per donor from 7 donors with T2D). No error bars are shown when less than 3 cells were analyzed per donor. A granule was defined as docked when the center of the granule was located within 150 nm from the plasma membrane.

(f) Whole-cell membrane conductance at 2.8 mM glucose in *Sox5*-kd and control cells (n=25-32 cells per group).

Data are mean ± SEM *p<0.05 using Student's t-test.

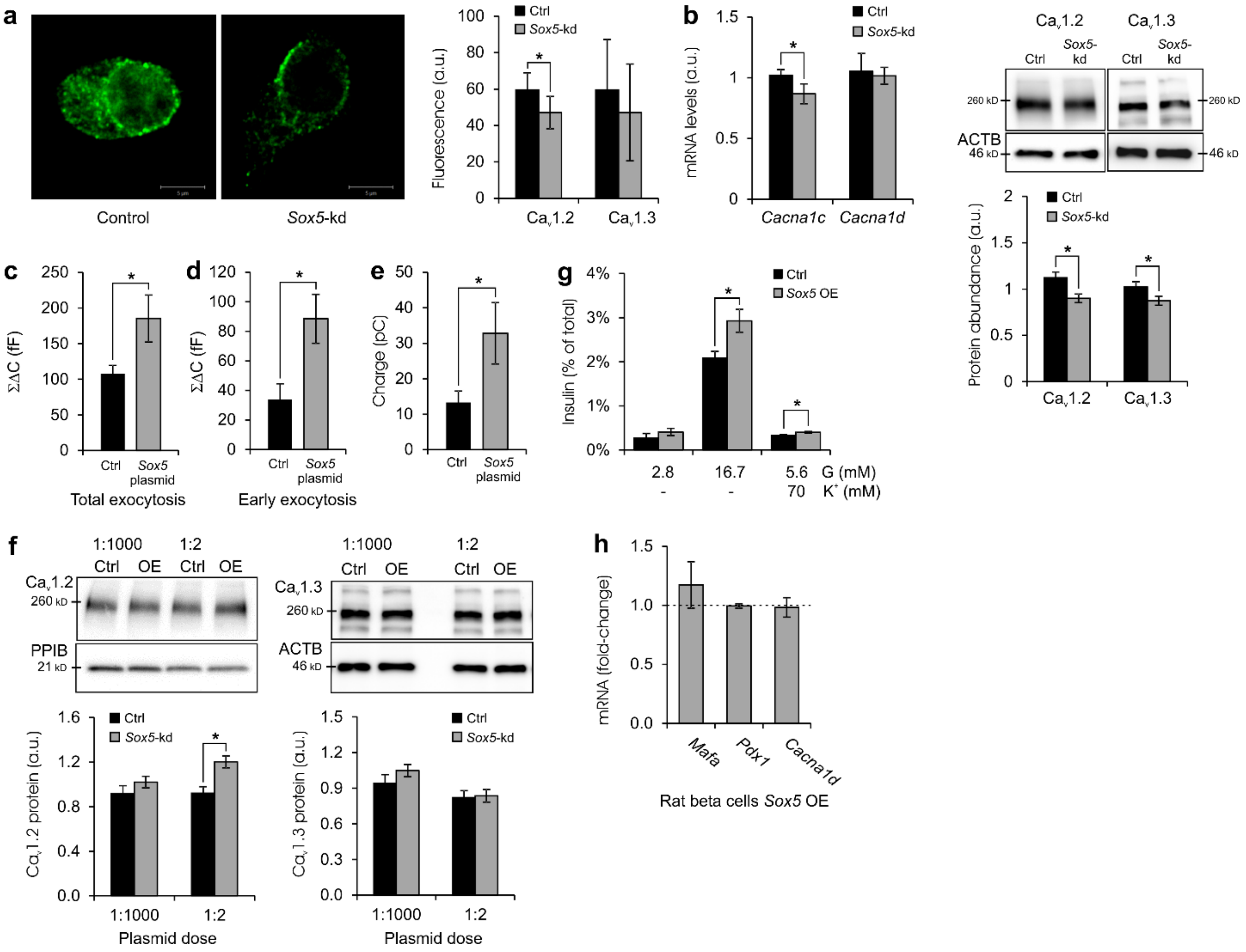

Supplementary Figure 5. Effects of knockdown and overexpression of *Sox5* on calcium channels and exocytosis

(a) Representative image of immunostaining of $Ca_V1.2$ in *Sox5*-kd cells and control (ctrl) INS-1 832/13 cells. Bar graphs show average expression determined by immunostaining for $Ca_V1.2$ and $Ca_V1.3$ in *Sox5*-kd cells and control cells (n=3 with 28 cells per condition in each experiment). Scale bar=5 µm.

(b) The bar graph to the left shows the mRNA levels of *Cacna1c* ($Ca_V1.2$) and *Cacna1d* ($Ca_V1.3$) in *Sox5*-kd and control INS-1 832/13 cells (n=4). To the right are representative immunoblots of total protein from *Sox5*-kd and control INS-1 832/13 cells. Polyclonal antibodies against $Ca_V1.2$, $Ca_V1.3$ and monoclonal antibody against β-actin (ACTB) were used as indicated. The graph shows average levels of $Ca_V1.2$ and $Ca_V1.3$ normalized to beta-actin in *Sox5*-kd and control cells (n=5-6).

(c) Total capacitance increase (ΣΔC) (`total exocytosis´) evoked by a train of ten 500 ms depolarizations from -70 to 0 mV applied to control INS-1 832/13 cells (n=17) and cells overexpressing *Sox5* (n=14 cells).

(d) Capacitance increase (ΣΔC) in response to the first two depolarizations of the train ('early exocytosis'). Data are from the same cells as in **(c)**.

(e) The integrated Ca^{2+}-current (charge) in the same cells as in **(c)**

(f) Representative immunoblots show $Ca_V1.2$ and $Ca_V1.3$ protein levels in control and *Sox5*-overexpressing cells (OE). The bars denote average protein levels of $Ca_V1.2$ and $Ca_V1.3$ normalized to cyclophilin B (PPIB) or beta-actin (ACTB) (n=5).
(g) mRNA expression of three genes in the β-cell fraction of sorted dispersed cells from Wistar rat islets after *Sox5* overexpression (OE) with lentivirus relative to islets transduced with control virus (n=3).
(h) Insulin secretion (as percent of total insulin content) from batch-incubated Wistar rat islets after *Sox5* overexpression with lentivirus relative to islets transduced with control virus. Insulin secretion was performed 72 h after transduction. Islets were incubated for 1 h with glucose (G) and for 15 min with K^+ as indicated (n=11 tubes per condition from 2 independent experiments).
Data are mean ± SEM *$p<0.05$ using Student's t-test.

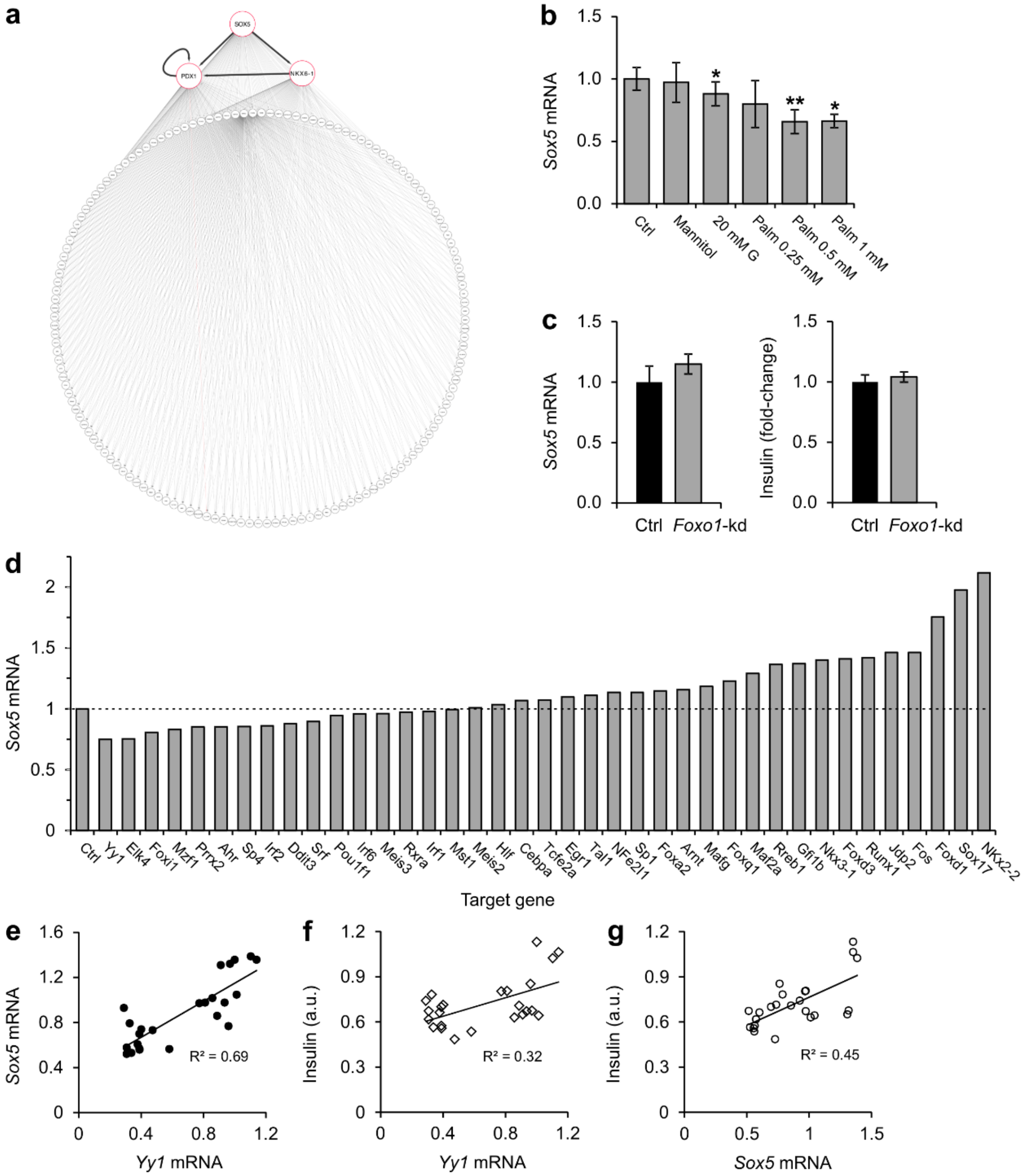

Supplementary Figure 6. Characterization of the regulation of Sox5

(a) The figure depicts a regulatory network generated by connecting *SOX5*, *PDX1* and *NKX6*.1 to open chromatin genes with motifs (in their promoter regions) for these three transcription factors. *SOX5*, *PDX1* and *NKX6.1* were significantly enriched for putative binding sites to the 168 open chromatin genes. Sox5 has predicted binding sites to 133 of the genes. Sox5 also has predicted binding sites to the promoter regions of *PDX1* and *NKX6.1*, and is thereby connected with 150 of the 168 genes in this regulatory network model.

(b) *Sox5* mRNA levels in INS-1 832/13 cells after 48 h incubation with 20 mM glucose (G), 20 mM mannitol and 0.25, 0.5 or 1 mM palmitate (palm). Data are normalized to the expression in non-treated cells (n=3).

(c) *Sox5* mRNA levels (left) and insulin secretion in response to 1 h stimulation at 16.7 mM glucose (right) in INS-1 832/13 cells treated with siRNA against *Foxo1* (*Foxo1*-kd) or negative control siRNA (Ctrl). Data are normalized to control (n=7).
(d) *Sox5* mRNA levels after transfection with siRNA targeting each of the 39 putative regulators of *Sox5* expression. Data are from triplicate measurements in one experiment.
(e) *Yy1* mRNA levels plotted against *Sox5* mRNA levels in *Yy1*-kd cells and control cells treated with 0, 0.25, 0.5 or 1 mM palmitate (graph shows pooled data from the different conditions; n=3).
(f) *Yy1* mRNA levels plotted against insulin secretion at 16.7 mM glucose. Data are from the same experiments as in **(e)**.
(g) *Sox5* mRNA levels plotted against insulin secretion at 16.7 mM glucose. Data as in **(e)**.
Data are mean ± SEM *p<0.05; **p<0.01 using Student's t-test.

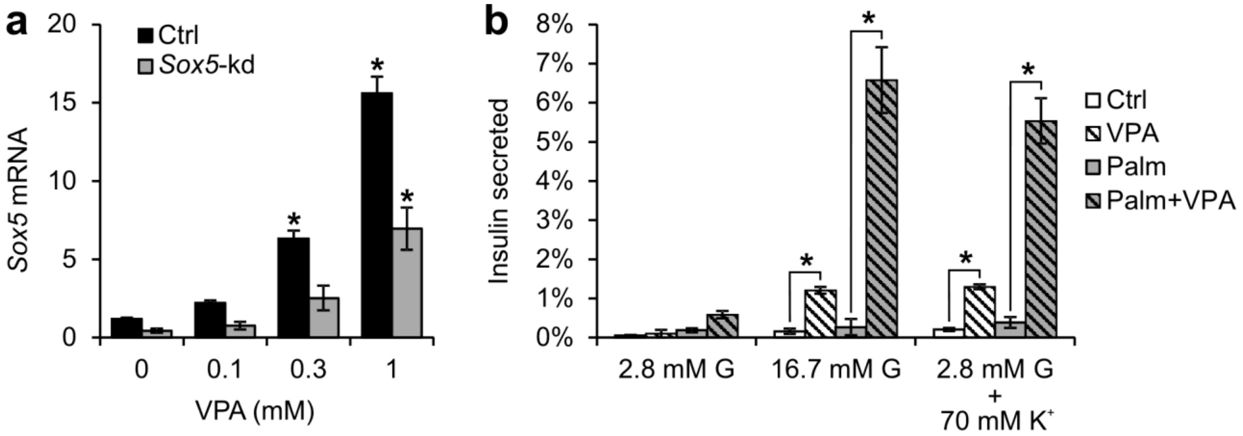

Supplementary Figure 7. Effect of valproic acid on *Sox5* expression and insulin secretion

(a) Sox5 mRNA levels in *Sox5*-kd and control cells (Ctrl) in response to valproic acid (VPA) at the doses indicated. *Sox5* levels in the presence of VPA relative to non-VPA-treated cells were used for statistical comparisons (n=4).

(b) Insulin secretion (as percent of total insulin) in islets from C57BL/6 male mice cultured with 1 mM VPA at 5 mM glucose with or without 1 mM palmitate for 72 h before measurements. Insulin secretion was measured after 1-h incubation with 2.8 and 16.7 mM glucose and after 15 min incubation with [2.8 mM glucose + 70 mM K^+] (n=3).

Data are mean ± SEM *p<0.05 using Student's t-test.

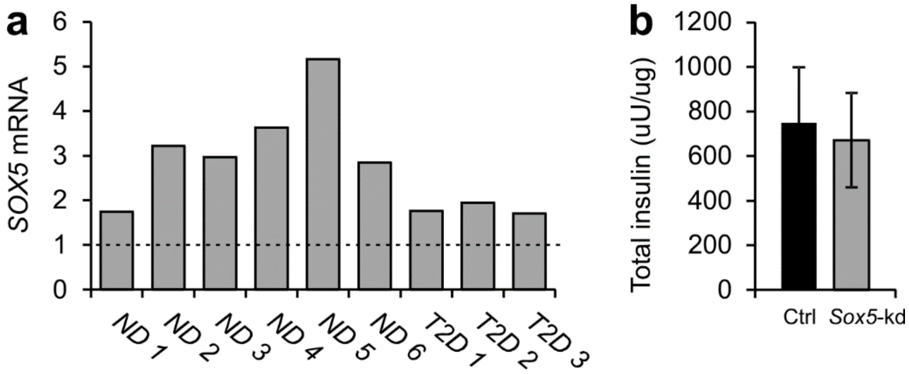

Supplementary Figure 8. *SOX5* overexpression in human islets

(a) *SOX5* overexpression in human islets after lentiviral transduction relative to islets transduced with control virus. Islets are from 6 non-diabetic donors (ND) and 3 diabetic donors (T2D).

(b) Insulin content of human islets with *SOX5* overexpression after lentiviral transduction relative to islets transduced with control virus (n=9 donors).

Data are mean ± SEM

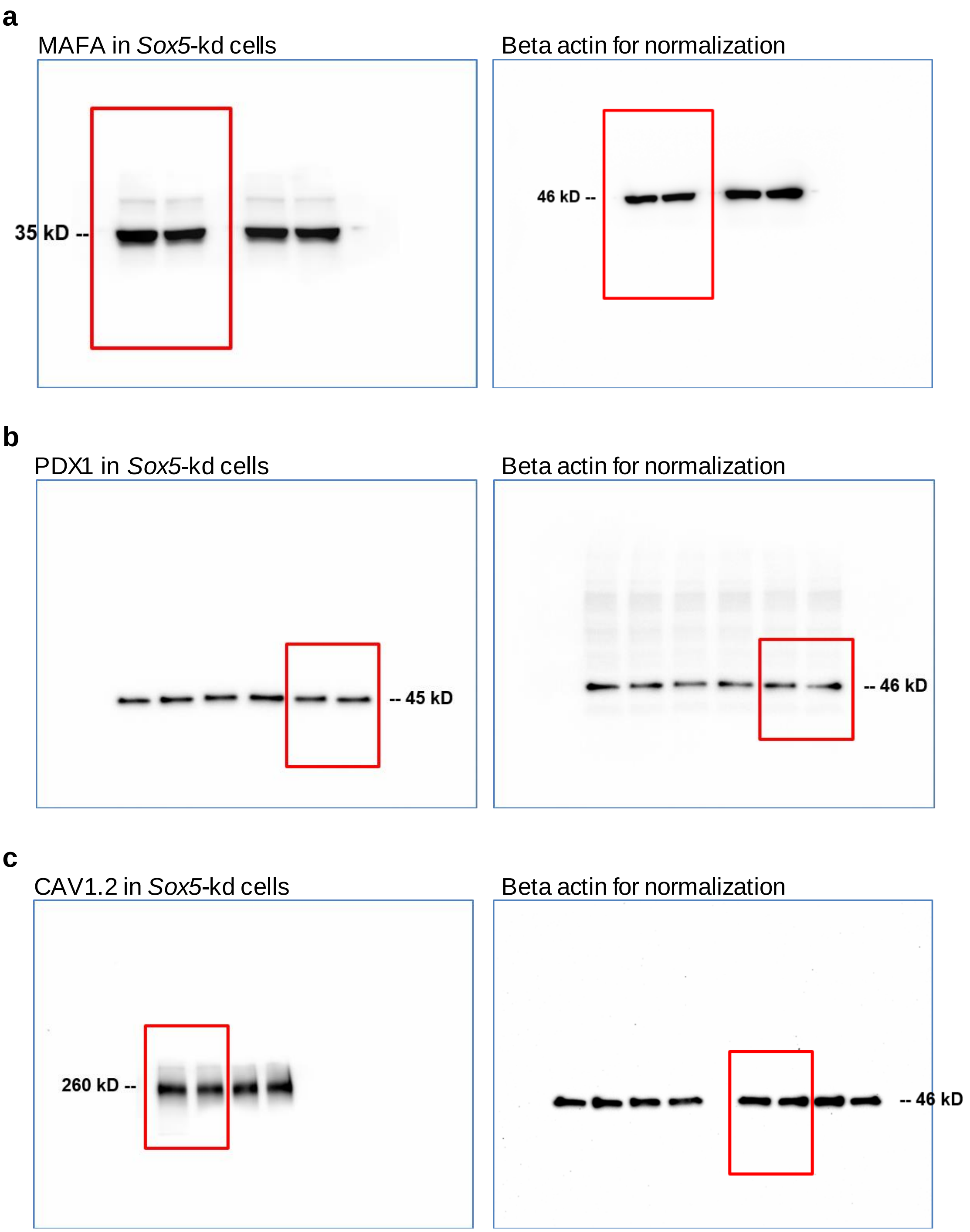
a
MAFA in Sox5-kd cells
35 kD --
Beta actin for normalization
46 kD --
b
PDX1 in Sox5-kd cells
-- 45 kD
Beta actin for normalization
-- 46 kD
c
CAV1.2 in Sox5-kd cells
260 kD --
Beta actin for normalization
-- 46 kD

d

CAV1.3 in *Sox5*-kd cells   Beta actin for normalization

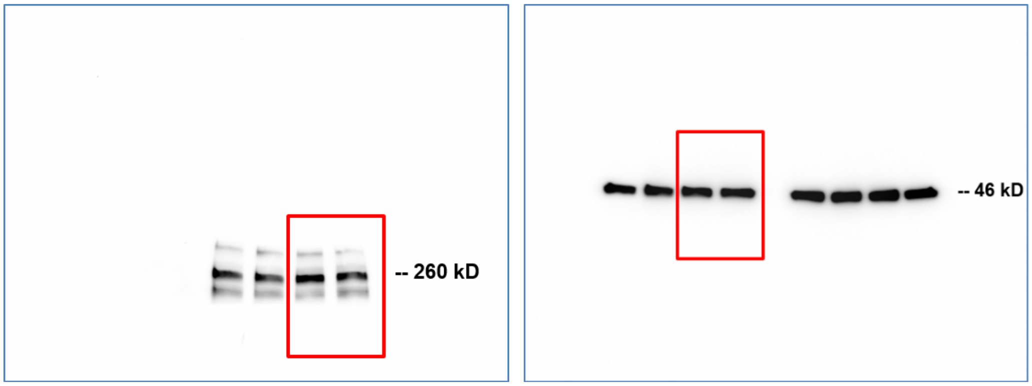

e

CAV1.2 in *Sox5*-overexpressing cells   Cyclophilin B for normalization

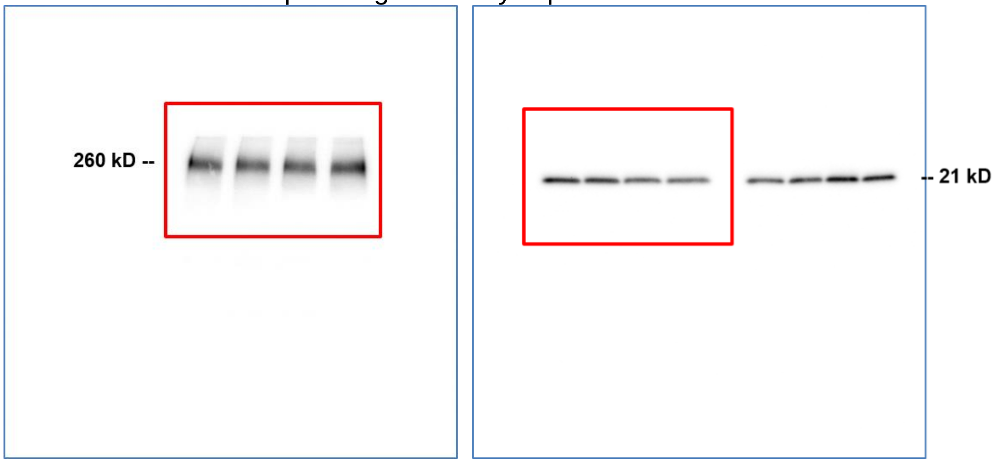

f

CAV1.3 in *Sox5*-overexpressing cells   Beta actin for normalization

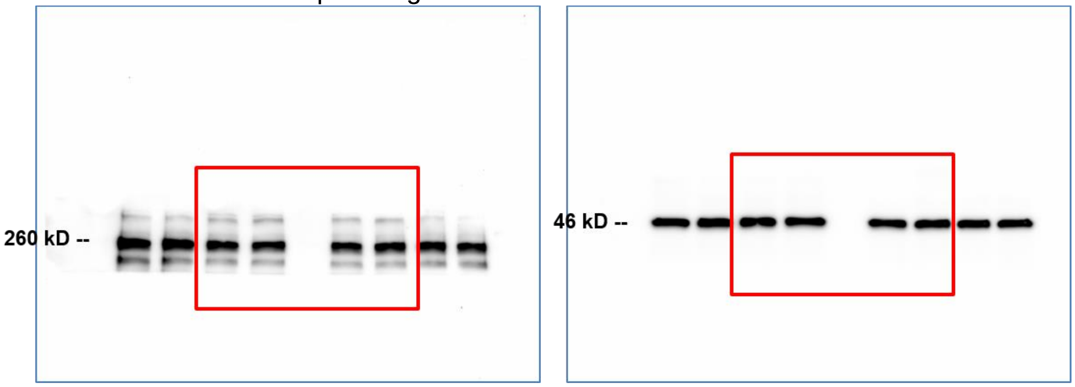

Supplementary Figure 9. Full pictures of protein blots displayed in Supplemental Figures
Protein blots from which the red boxes correspond, with similar band order, to Supplemental Figure 3b **(a-b)**, Supplemental Figure 5b **(c-d)**, and Supplemental Figure 5f **(e-f)**.
(a) MAFA (left) and beta actin for normalization (right) in control and INS-1 832/13 cells with *Sox5*-kd.

(b) PDX1 (left) and beta actin for normalization (right) in control and INS-1 832/13 cells with *Sox5*-kd.
(c) CAV1.2 (left) and beta actin for normalization (right) in control and INS-1 832/13 cells with *Sox5*-kd.
(d) CAV1.3 (left) and beta actin for normalization (right) in control and INS-1 832/13 cells with *Sox5*-kd.
(e) CAV1.2 (left) and cyclophilin B for normalization (right) in control and INS-1 832/13 cells overexpressing *Sox5*.
(f) CAV1.3 (left) and beta actin for normalization (right) in control and INS-1 832/13 cells overexpressing *Sox5*.

Supplementary Table 1. Characteristics of human islet donors

	All (n=64)	Non-diabetic (n=45)	T2D (n=19)
Age (years)	57 ± 10	56 ± 11	59 ± 9
Sex (female / male)	33 / 31	23 / 22	10 / 9
BMI (kg m^{-2})	26.2 ± 3.8	25.2 ± 2.9	28.7 ± 4.6
Purity (%)	66±18	69±17	61±20
Number of donors from which HbA1c was available	52	34	18
Number of donors from which insulin secretion measurements were conducted	48	32	16

Data are presented as mean ± s.d.

Supplementary Table 2. Analysis of network properties

β	R^2	mean *k*	max *k*
1	0.00904	4820	7990
2	0.351	1290	3160
3	0.630	448	1660
4	0.750	185	1020
5	0.811	86.9	685
6	0.834	45.0	488
7	0.851	25.2	363
8	0.876	15.1	279
9	0.868	9.50	220
10	0.878	6.26	177
11	0.887	4.28	144
12	0.902	3.03	119

Values of β from 1 to 12 were used to generate the adjacency matrix $a_{i,j} = |cor(x_i,x_j)|^{\beta}$, where x_i represents the expression vector for gene i across the 64 samples. The degree *k* denotes the connectivity of a gene. A linear relation between the logarithm of k and the logarithm of the degree distribution p*(k)* implies that the network has scale-free topology. R^2 denotes the scale-free fit index. To obtain a network with approximately scale-free topology (R^2>0.8) and yet high connectivity, β=5 was used for subsequent analyses.

Supplementary Table 3. Association of module eigengenes with phenotype traits

Module	No of genes	T2D (n=64)		HbA1c (n=52)		GSIS (n=48)		KSIS (n=26)	
		p-value	beta	p-value	beta	p-value	beta	p-value	beta
1	4328	0.58	0.04	0.77	-0.049	0.22	-0.17	0.33	-0.12
**2**	**3148**	**0.013**	**-0.32**	**0.0025**	**-0.39**	**0.0059**	**0.37**	**0.048**	**0.41**
3	1474	0.53	-0.032	0.94	-0.035	0.73	-0.13	0.27	-0.31
4	1223	0.52	0.06	0.93	0.043	0.64	0.039	0.94	-0.13
5	1116	0.52	0.079	0.16	0.25	0.34	-0.14	0.79	-0.019
6	815	0.011	0.3	0.031	0.32	0.1	-0.24	0.32	-0.4
7	806	0.39	0.071	0.57	-0.036	0.12	-0.2	0.25	-0.22
8	714	0.16	0.22	0.087	0.21	0.46	-0.2	0.32	-0.092
9	702	0.014	-0.3	0.0097	-0.33	0.0087	0.33	0.11	0.38
10	649	0.4	-0.11	0.35	-0.15	0.2	0.21	0.24	0.18
11	598	0.034	-0.25	0.0088	-0.34	0.0031	0.36	0.089	0.38
12	424	0.037	-0.26	0.026	-0.25	0.018	0.33	0.058	0.38
13	380	0.56	0.15	0.54	0.17	0.65	-0.1	0.64	-0.14
14	358	0.00055	0.39	0.0041	0.38	0.033	-0.25	0.22	-0.43
15	347	0.18	-0.088	0.33	-0.063	0.61	0.087	0.99	-0.12
16	209	0.48	0.038	0.16	0.1	0.69	-0.014	0.92	0.033
17	203	0.07	-0.23	0.24	-0.099	0.063	0.22	0.1	0.4
18	198	0.67	0.12	0.69	0.069	0.69	-0.032	0.83	-0.054
19	196	0.0064	0.32	0.002	0.41	0.0061	-0.35	0.19	-0.34
20	196	0.43	0.14	0.89	0.087	0.27	0.19	0.086	0.18
21	188	0.53	-0.088	0.6	-0.097	0.1	0.21	0.34	0.21
22	159	0.12	-0.19	0.48	-0.019	0.3	0.16	0.35	0.28
23	152	0.17	0.19	0.13	0.29	0.97	-0.072	0.073	0.061
24	151	0.13	-0.18	0.23	-0.18	0.4	0.17	0.62	0.05
25	143	0.77	0.094	0.27	0.19	0.35	-0.13	0.24	-0.28
26	140	0.024	0.23	0.06	0.21	0.15	-0.12	0.33	-0.27
27	125	0.056	-0.21	0.42	-0.12	0.48	0.05	0.46	0.23
28	120	0.043	-0.26	0.31	-0.15	0.36	0.081	0.68	0.087
29	115	0.89	0.025	0.96	0.032	0.075	0.2	0.19	0.23
30	112	0.7	0.00093	0.5	-0.079	0.44	0.19	0.077	0.21
31	95	0.04	0.27	0.13	0.26	0.82	-0.1	0.97	-0.043
32	70	0.75	0.071	0.26	0.23	0.38	-0.099	0.62	-0.067
33	65	0.31	0.094	0.011	0.21	0.41	-0.051	0.83	0.0038
34	64	0.57	0.11	0.2	0.21	0.87	-0.06	0.97	-0.071
35	64	0.013	-0.31	0.024	-0.3	0.011	0.29	0.36	0.33
36	64	0.31	-0.14	0.59	-0.081	0.44	0.075	0.38	0.29
37	58	0.38	-0.16	0.65	-0.048	0.4	0.13	0.39	0.27
38	51	0.15	-0.13	0.037	-0.18	0.0043	0.31	0.089	0.33
39	50	0.43	-0.079	0.55	-0.2	0.34	-0.17	0.11	-0.34

40	50	0.56	0.11	0.54	0.18	0.32	0.15	0.22	0.29
41	44	0.69	-0.0083	0.75	0.011	0.22	0.16	0.95	-0.013
42	43	0.1	0.22	0.66	0.14	0.17	0.024	0.96	0.084
43	40	0.3	-0.12	0.47	-0.19	0.2	-0.24	0.077	-0.44
44	38	0.42	0.068	0.89	0.037	0.75	0.046	0.47	0.13
45	35	0.59	0.044	0.86	-0.02	0.91	-0.055	0.95	0.13
46	32	0.52	-0.04	0.11	-0.087	0.7	0.014	0.99	-0.17
47	31	0.18	0.16	0.41	0.23	0.97	0.034	0.15	0.24
48	30	0.2	0.095	0.15	0.076	0.65	-0.14	0.95	0.022
49	26	0.53	-0.027	0.72	0.14	0.03	0.25	0.43	0.29
50	24	0.8	0.012	0.8	-0.065	0.056	-0.24	0.91	-0.034
51	24	0.71	0.012	0.66	-0.022	0.9	0.041	0.92	-0.12
52	21	0.54	-0.092	0.12	-0.36	0.99	-0.045	0.056	-0.35
53	20	0.083	-0.18	0.0093	-0.3	0.019	0.3	0.17	0.2
54	17	0.55	0.064	0.48	0.17	0.16	0.094	0.92	-0.068
55	17	0.82	0.0065	0.55	0.2	0.076	0.21	0.24	0.2
56	16	0.38	0.13	0.64	0.16	0.19	0.18	0.28	0.24

The module eigengene denotes the 1st principal component of the expression matrix of the module genes across the 64 samples. For each module eigengene we analyzed the association with T2D status, HbA1c, glucose-stimulated insulin secretion (GSIS) and K^+-stimulated insulin secretion (KSIS). The T2D-associated module is indicated in boldface. P-values shown in the table are not corrected for multiple testing.

Supplementary Table 4. **Module genes with islet-selective open chromatin**

Gene symbol	Fold-change in T2D	Connectivity (k_{in})
ABCC9	-1.13	225
ABHD10	-1.19	345
ACVR1C	-1.28	293
ADAMTS2	-1.13	128
AGBL4	-1.10	92
ANKS1B	-1.04	137
ATRNL1	-1.27	330
BARX2	1.12	150
BMP5	-1.24	172
C1orf158	1.01	15
C1orf168	-1.01	90
C3orf14	-1.11	222
C9orf150	1.03	77
CACNA1H	-1.11	92
CACNA2D1	-1.23	364
CACNB2	-1.14	278
CADM1	-1.14	380
CADPS	-1.21	316
CERKL	-1.13	403
CHODL	-1.10	61
CNTN1	-1.19	498
CNTN4	-1.33	245
CPE	-1.16	474
D4S234E	-1.26	314
DACH1	-1.16	459
DCX	-1.13	200
DGKB	-1.05	51
DIRAS2	-1.15	118
DSCAM	-1.01	45
DZIP3	-1.21	364
ELAVL4	-1.29	420
ELP4	-1.20	213
EML5	-1.17	155
ENAM	-1.11	174
FAM123C	-1.06	85
FAM135A	-1.15	50
FAM135B	-1.06	247
FAM148A	-1.14	20
FAM19A4	-1.17	81
FAM84A	-1.03	27

FBXW7	-1.07	105
FGF14	-1.19	475
FLJ25770	-1.17	148
FOXC1	1.03	69
FOXP2	-1.06	180
G6PC2	-1.31	263
GABRA1	-1.19	19
GABRB3	-1.13	431
GABRG2	-1.25	209
GAD2	-1.19	283
GDAP1	-1.17	338
GLP1R	-1.35	262
GNAI1	-1.10	394
GPM6A	-1.30	99
GPR27	-1.05	76
GRIA2	-1.24	288
GRIA4	-1.30	152
GRIK2	-1.10	286
HMGCLL1	-1.22	377
HS6ST3	-1.18	330
IAPP	-1.38	68
IGSF11	-1.14	246
ISL1	-1.19	223
JAKMIP2	-1.14	382
KBTBD7	-1.19	241
KCNB2	-1.21	327
KCNK16	-1.23	226
KCNMA1	-1.18	415
KIAA0774	-1.19	249
KIAA1257	-1.04	83
KIAA1383	-1.03	37
KIAA1486	-1.07	56
KIAA1804	1.08	102
KL	-1.13	360
KLHL1	-1.18	43
LIG4	-1.08	140
LIMCH1	-1.11	255
LRRTM3	-1.33	249
LSAMP	1.01	62
MAGI2	-1.16	335
MAP9	-1.04	171
MAPK10	-1.15	367
MCTP2	-1.11	41

MEIS2	-1.22	309
MEIS3	-1.02	28
MMP16	-1.02	57
MS4A8B	-1.17	89
MYO3A	-1.16	269
NALCN	-1.19	443
NBEA	-1.19	272
NECAB2	-1.01	89
NEGR1	1.02	156
NEUROD1	-1.22	382
NKX6-1	-1.27	283
NMNAT3	-1.05	118
NOL4	-1.24	482
NR3C2	-1.11	94
NRXN1	-1.15	473
ODZ3	-1.08	256
OXGR1	-1.10	161
PAM	-1.19	411
PAPPA2	-1.10	88
PAX6	-1.20	337
PCDH17	-1.13	349
PCDH20	-1.12	74
PCDHAC1	-1.06	269
PCLO	-1.22	300
PCP4	-1.21	81
PDK4	-1.04	131
PDX1	-1.21	129
PDZRN3	-1.09	295
PGR	-1.12	355
PRKACB	-1.14	345
PRUNE2	-1.08	43
PVRL3	-1.21	277
RAB3C	-1.17	311
RALYL	-1.08	70
RASEF	1.27	373
RGS17	-1.16	108
RGS4	-1.10	202
RIMBP2	-1.23	360
RIMS2	-1.18	342
RNF150	-1.08	245
RNF180	-1.12	250
ROBO2	-1.35	300
ROR1	-1.07	111

RORB	-1.12	222
RTN1	-1.15	426
SCG2	-1.17	372
SCGB2A1	-1.07	42
SDK1	-1.02	143
SERPINI2	1.08	72
SGCB	-1.08	216
SGCZ	-1.03	58
SIM1	-1.16	279
SLC17A6	-1.26	176
SLC18A2	-1.21	61
SLC30A8	-1.20	259
SLC35F4	-1.12	90
SLC5A1	-1.07	62
SLC5A8	-1.03	39
SLC7A2	-1.08	195
SLCO1A2	-1.22	80
SNAP91	-1.24	455
SOX6	-1.14	125
SPATA7	-1.12	100
SPTB	-1.10	260
ST18	-1.27	345
ST6GALNAC5	-1.23	286
ST8SIA3	-1.12	395
STXBP5L	-1.25	380
SULT4A1	-1.14	131
SYT14	-1.27	431
SYT4	-1.24	459
TMEM132B	-1.19	58
TMEM132D	-1.09	242
TMEM196	-1.21	516
TMEM61	-1.07	161
TMEM63C	-1.24	490
TRPM3	-1.24	338
TSC22D1	-1.05	106
TSHZ3	-1.10	84
TSPYL5	-1.11	276
TTC8	-1.16	450
UNC5D	-1.17	47
ZNF223	-1.09	80
ZNF585B	-1.10	217
ZSCAN18	-1.06	99

Fold-change denotes mRNA expression in T2D donors vs. non-diabetic donors.

Supplementary Table 5. Characteristics of human islet donors in replication set

	All (n=59)	Non-diabetic (n=37)	T2D (n=22)
Age (years)	61 ± 10	64 ± 7	60 ± 12
Sex (female / male)	23 / 36	17 / 20	6 / 16
BMI (kg m^{-2})	26.8 ± 3.8	26.4 ± 3.7	27.4 ± 3.9
Purity (%)	72 ± 18	72 ± 17	72 ± 21
Number of donors from which HbA1c was available	57	35	22

Data are presented as mean ± s.d. No insulin secretion data were available from the replication set.

Supplementary Table 6. Analysis of network properties in replication set

b	R^2	mean *k*	max *k*
1	0.089	2030	3233
2	0.192	653	1510
3	0.459	266	838
4	0.601	126	516
5	0.682	66	340
6	0.730	38	235
7	0.756	23	169
8	0.800	14	126
9	0.803	9	96
10	0.767	6	74
11	0.817	4	58
12	0.834	3	47

Values of b from 1 to 12 were used to generate the adjacency matrix $a_{i,j} = |cor(x_i,x_j)|^b$, where x_i represents the expression vector for gene i across the 59 samples in the replication set. The degree *k* denotes the connectivity of a gene. A linear relation between the logarithm of *k* and the logarithm of the degree distribution p(*k*) implies that the network has scale-free topology. R^2 denotes the scale-free fit index. To obtain a network with approximately scale-free topology (R^2>0.8) and yet high connectivity, b=8 was used for subsequent analyses.

Supplementary Table 7. Module genes with islet-selective open chromatin in replication set

Gene Symbol	Fold-change in T2D
ABCC9	-1.13
ABHD10	-1.10
ACVR1C	-1.28
AGBL4	-1.08
ANKS1B	-1.06
ATRNL1	-1.10
BARX2	1.04
BMP5	-1.01
C1orf158	-1.08
C1orf168	1.00
C3orf14	-1.13
CACNA1H	-1.02
CACNA2D1	-1.09
CACNB2	-1.03
CADM1	-1.03
CADPS	-1.15
CERKL	-1.08
CHODL	-1.12
CNTN1	-1.13
CNTN4	-1.27
CPE	-1.05
DACH1	-1.06
DCX	-1.13
DGKB	1.06
DIRAS2	-1.14
DSCAM	-1.04
DZIP3	-1.11
ELAVL4	-1.07
ELP4	-1.10
EML5	-1.07
ENAM	-1.10
FAM123C	-1.01
FAM135A	-1.10
FAM135B	1.04
FAM19A4	-1.11
FAM84A	-1.16
FBXW7	-1.07
FGF14	-1.06
FOXC1	1.08

FOXP2	-1.06
G6PC2	-1.16
GABRA1	-1.31
GABRB3	-1.14
GABRG2	-1.38
GAD2	-1.04
GDAP1	-1.13
GLP1R	-1.15
GNAI1	-1.01
GPM6A	-1.11
GRIA2	-1.11
GRIA4	-1.05
GRIK2	-1.21
HMGCLL1	-1.12
HS6ST3	-1.15
IAPP	-1.13
IGSF11	-1.05
ISL1	-1.24
JAKMIP2	1.03
KBTBD7	-1.22
KCNB2	-1.15
KCNK16	-1.11
KCNMA1	-1.11
KIAA1383	-1.01
KL	-1.16
KLHL1	-1.30
LIG4	-1.05
LIMCH1	-1.03
LRRTM3	-1.20
LSAMP	1.08
MAGI2	-1.14
MAP9	-1.04
MAPK10	-1.07
MCTP2	-1.09
MEIS2	-1.12
MEIS3	-1.02
MMP16	-1.21
MS4A8B	-1.22
MYO3A	-1.03
NALCN	-1.05
NBEA	-1.11
NECAB2	1.04
NEGR1	1.00

NEUROD1	-1.16
NKX6-1	-1.05
NMNAT3	-1.14
NOL4	-1.16
NR3C2	-1.00
NRXN1	-1.10
ODZ3	-1.07
OXGR1	-1.03
PAM	-1.06
PAPPA2	1.07
PAX6	-1.13
PCDH17	-1.07
PCDH20	-1.35
PCDHAC1	-1.02
PCLO	-1.06
PCP4	-1.16
PDX1	-1.22
PDZRN3	-1.07
PGR	-1.10
PRKACB	-1.10
PRUNE2	-1.06
PVRL3	-1.05
RAB3C	-1.22
RALYL	-1.18
RIMBP2	-1.16
RIMS2	-1.10
RNF150	-1.05
RNF180	-1.02
ROBO2	-1.16
ROR1	1.01
RORB	-1.12
RTN1	-1.11
SCG2	-1.04
SCGB2A1	1.05
SDK1	-1.04
SGCB	-1.07
SGCZ	-1.08
SIM1	-1.12
SLC17A6	1.01
SLC18A2	1.13
SLC30A8	-1.14
SLC35F4	-1.21
SLC5A1	-1.08

SLC7A2	-1.13
SLCO1A2	1.01
SOX6	-1.11
SPATA7	-1.10
SPTB	1.01
ST18	-1.19
ST6GALNAC5	-1.17
ST8SIA3	-1.02
STXBP5L	-1.16
SULT4A1	-1.00
SYT14	-1.15
SYT4	-1.11
TMEM132B	-1.13
TMEM132D	-1.11
TMEM196	-1.10
TMEM61	-1.07
TMEM63C	-1.11
TRPM3	-1.14
TSC22D1	-1.05
TSHZ3	-1.04
TSPYL5	-1.14
TTC8	-1.07
UNC5D	-1.15
ZNF585B	-1.06
ZSCAN18	-1.06

Fold-change denotes mRNA expression in T2D donors vs. non-diabetic donors.

Supplementary Table 8. SNPs associated with module eigengenes

SNP id	Nearest gene	Associated trait	P value
rs4406574	*SMARCA1*	Eigengene T2D module	1.42E-04
rs5975111	*SMARCA1*	Eigengene T2D module	1.99E-04
rs5932625	*SMARCA1*	Eigengene T2D module	1.42E-04
rs11796659	*SMARCA1*	Eigengene T2D module	1.99E-04
rs5932624	*SMARCA1*	Eigengene T2D module	2.19E-04
rs5977087	*SMARCA1*	Eigengene T2D module	1.99E-04
rs1408301	*SMARCA1*	Eigengene T2D module	1.99E-04
rs10505917	*SOX5*	Eigengene T2D module	1.28E-03
rs7314161	*SOX5*	Eigengene T2D module	1.19E-03
rs11047351	*SOX5*	Eigengene 168 genes	1.67E-03
rs17498948	*SOX5*	Eigengene 168 genes	1.67E-03
rs4569092	*SOX5*	Eigengene 168 genes	1.15E-03
rs4963749	*SOX5*	Eigengene 168 genes	1.67E-03

Associations between donor genotype (n=64 donors) and the eigengenes of the T2D-related module and the 168 open chromatin genes, respectively, were analyzed by linear regression using age, sex and BMI as covariates. The nearest genes to each SNP and the p-values from the linear regression are shown.

Supplementary Table 9. Direction of change of signature genes in INS-1 832/13 cells with Sox5 knockdown or Sox5 overexpression

Sox5-kd:<br>*Sox5* OE:	Same as in T2D<br>Opposite to T2D<br>(n=50)	Same as in T2D<br>No change<br>(n=28)	No change<br>Opposite to T2D<br>(n=37)	No change<br>No change<br>(n=24)
	ABCC9	*ABHD10*	*ACVR1C*	*CHODL*
	ADAMTS2	*AGBL4*	*CACNB2*	*DIRAS2*
	BARX2	*ANKS1B*	*CADM1*	*DSCAM*
	BMP5	*ATRNL1*	*CNTN1*	*FOXC1*
	CACNA1H	*DCX*	*CPE*	*MAGI2*
	CACNA2D1	*DGKB*	*EML5*	*MAPK10*
	CADPS	*ELAVL4*	*FAM135B*	*MEIS3*
	CNTN4	*FAM19A4*	*GABRA1*	*MMP16*
	DZIP3	*FGF14*	*GAD2*	*MYO3A*
	ENAM	*GABRB3*	*GLP1R*	*NECAB2*
	FAM135A	*GRIK2*	*GNAI1*	*NEGR1*
	FAM84A	*JAKMIP2*	*GPM6A*	*NKX6-1*
	FOXP2	*KCNB2*	*HS6ST3*	*PAM*
	GABRG2	*KCNK16*	*IAPP*	*PCDH20*
	GDAP1	*LRRTM3*	*IGSF11*	*PCP4*
	GPR27	*LSAMP*	*KLHL1*	*PDK4*
	GRIA2	*NALCN*	*MCTP2*	*PDX1*
	GRIA4	*NMNAT3*	*MEIS2*	*PRUNE2*
	HMGCLL1	*NR3C2*	*NRXN1*	*RNF150*
	ISL1	*PGR*	*OXGR1*	*SGCZ*
	KCNMA1	*PVRL3*	*PCDH17*	*SULT4A1*
	KL	*RALYL*	*PDZRN3*	*SYT14*
	LIG4	*RORB*	*RGS4*	*TSPYL5*
	LIMCH1	*SLC18A2*	*RNF180*	*ZSCAN18*
	MAP9	*ST18*	*ROR1*	
	NEUROD1	*ST8SIA3*	*RTN1*	
	NOL4	*TMEM63C*	*SCG2*	
	PAX6	*TSHZ3*	*SLC30A8*	
	PCLO		*SLC35F4*	
	PRKACB		*SLCO1A2*	
	RAB3C		*SOX6*	
	RASEF		*ST6GALNAC5*	
	RGS17		*STXBP5L*	
	RIMBP2		*SYT4*	
	RIMS2		*TMEM132D*	
	ROBO2		*TRPM3*	
	SCGB2A1		*TSC22D1*	

SERPINI2
SGCB
SIM1
SLC17A6
SLC5A1
SLC5A8
SLC7A2
SNAP91
SPATA7
SPTB
TMEM132B
TMEM196
TTC8

INS-1 832/13 cells were transfected with Sox5 siRNA (*Sox5*-kd) or a Sox5 plasmid (*Sox5* OE). The table displays genes in the T2D-associated module that exhibited a statistically significant change in expression in the same or opposite direction to that observed in T2D vs. non-diabetic islets or no change.

Supplementary Table 10. Genes involved in glucose metabolism that are affected in response to *Sox5*-knockdown

Gene symbol	Gene name	Microarray Fold-change	Microarray P-value	qPCR Fold-change	qPCR P-value
Cs	Citrate synthase	-1.21	0.0480	-1.03	0.614
Fh	Fumarate hydratase	-1.31	0.00500	-1.34	0.00613
Suclg2	Succinate-CoA ligase, GDP-forming, beta subunit	-1.24	0.00130	-1.30	0.00587
Sucla2	Succinate-CoA ligase, ADP-forming, beta subunit	-1.70	0.000300	-1.71	0.000421
Pdha1	Pyruvate dehydrogenase (lipoamide) alpha 1	-1.52	0.00120	-1.60	0.00209
Ldhb	Lactate dehydrogenase B	1.34	0.00300	1.48	0.0396
Ndufa10	NADH dehydrogenase (ubiquinone) 1 alpha subcomplex 10	-1.53	3.80E-05	-1.39	0.000983
Ndufv3	NADH dehydrogenase (ubiquinone) flavoprotein 3	-1.37	0.0410	-1.19	0.139
Sdha	Succinate dehydrogenase complex, subunit A, flavoprotein (Fp)	-1.44	4.90E-05	-1.65	0.00791
Sdhc	Succinate dehydrogenase complex, subunit C, integral membrane protein	-2.08	0.000200	-2.26	0.00102
Atp5f1	ATP synthase, H+ transporting, mitochondrial Fo complex, subunit B1	-1.92	0.000500	-2.48	0.00437

Genes encoding enzymes that are involved in glucose metabolism were analyzed in the microarrays and changes in expression were also analyzed by qPCR. Fold-change denotes differential gene expression in *Sox5*-kd cells vs. control cells (n=3-5).

Supplementary Table 11. Signature genes with changed expression in INS-1 832/13 cells after VPA treatment or Sox5 overexpression

Same direction of change with VPA and Sox5 OE (n=41)	Genes changed with VPA but not with Sox5 OE (n=38)
ADAMTS2	*ACVR1C*
AGBL4	*CADM1*
C1orf168	*CPE*
C3orf14	*DACH1*
CACNA1H	*DCX*
CADPS	*DSCAM*
CNTN1	*ELP4*
CNTN4	*EML5*
ELAVL4	*FAM135B*
FAM123C	*FAM84A*
GABRG2	*FLJ25770*
GRIA2	*GABRA1*
GRIK2	*GABRB3*
KBTBD7	*GPM6A*
KCNB2	*HMGCLL1*
KIAA0774	*IGSF11*
KIAA1486	*ISL1*
KL	*KCNMA1*
MEIS3	*KIAA1383*
NALCN	*KIAA1804*
NBEA	*KLHL1*
NEUROD1	*MAPK10*
NKX6-1	*MMP16*
NOL4	*MYO3A*
NRXN1	*NR3C2*
PCDHAC1	*OXGR1*
PDK4	*PAM*
RGS4	*PAPPA2*
RIMBP2	*PCLO*
RNF180	*PRKACB*
SLC17A6	*PRUNE2*
SLC18A2	*RNF150*
SLC30A8	*ROR1*
SLC7A2	*RTN1*
SLCO1A2	*SOX6*
ST6GALNAC5	*TMEM196*
ST8SIA3	*TSHZ3*

SYT14	*ZSCAN18*
TMEM132B	
TRPM3	
TTC8	

Genes in the T2D signature that were significantly changed in response to treatment with 1 mM VPA or transfection with a Sox5 plasmid (Sox5 OE) relative to control INS-1 832/13 cells (n=3).

Supplementary Table 12. Association between T2D-associated risk variants and gene expression in human islets

SNP		Eigengene for T2D-associated module	Eigengene for the 168 genes (T2D signature)	SOX5 expression
KCNQ1_rs2237895	Effect size	0.0702	0.133	0.0588
	p-value	0.581	0.295	0.644
	N	64	64	64
MTNR1B_rs10830963	Effect size	-0.0002	0.000	-0.0052
	p-value	0.999	1.000	0.968
	N	64	64	64
ADRA2A_rs553668	Effect size	-0.0724	-0.020	0.0152
	p-value	0.573	0.874	0.906
	N	63	63	63
CDKAL1_rs7754840	Effect size	0.0932	-0.098	-0.0438
	p-value	0.464	0.441	0.731
	N	64	64	64
TCF7L2_rs7903146	Effect size	-0.0449	-0.179	-0.1336
	p-value	0.731	0.167	0.305
	N	61	61	61
GIPR_rs10423928	Effect size	0.0569	0.272	0.2329
	p-value	0.658	0.313	0.066
	N	63	63	63
IGF2BP2_rs4402960	Effect size	-0.1701	-0.150	-0.1195
	p-value	0.179	0.236	0.347
	N	64	64	64
SLC30A8_rs13266634	Effect size	-0.0153	0.104	0.1784
	p-value	0.911	0.445	0.188
	N	56	56	56
CAMK1D_rs12779790	Effect size	-0.1775	-0.093	-0.1242
	p-value	0.164	0.469	0.332
	N	63	63	63
FTO_rs9939609	Effect size	-0.0268	-0.077	-0.0249
	p-value	0.834	0.547	0.845
	N	64	64	64

HHEX_rs1111875	Effect size	0.2088	0.134	0.1193
	p-value	0.098	0.291	0.348
	N	64	64	64
KCNJ11_rs5219	Effect size	-0.0639	-0.114	-0.1444
	p-value	0.616	0.371	0.255
	N	64	64	64
TSPAN8_LGR5_rs7961581	Effect size	0.0730	0.067	0.1082
	p-value	0.567	0.600	0.395
	N	64	64	64
JAZF1_rs864745	Effect size	0.0857	0.029	0.0591
	p-value	0.504	0.820	0.645
	N	63	63	63
WFS1_10010131	Effect size	-0.0490	-0.056	-0.0898
	p-value	0.707	0.670	0.491
	N	61	61	61
CDKN2A2B_rs10811661	Effect size	-0.0085	-0.025	-0.0778
	p-value	0.948	0.848	0.545
	N	63	63	63
PPARG_rs1801282	Effect size	0.0070	0.022	0.0181
	p-value	0.956	0.861	0.887
	N	64	64	64
NOTCH2_rs10923931	Effect size	0.1628	-0.013	-0.0023
	p-value	0.199	0.921	0.986
	N	64	64	64
ADAMTS9_rs4607103	Effect size	-0.0095	0.173	0.2245
	p-value	0.941	0.172	0.075
	N	64	64	64
G6PC2_rs560887	Effect size	-0.0019	0.157	0.1471
	p-value	0.988	0.214	0.246
	N	64	64	64
GCK_rs4607517	Effect size	0.0348	-0.012	-0.0387
	p-value	0.790	0.929	0.767
	N	61	61	61
GCKR_rs780094	Effect size	0.2438	0.253	0.3570

	p-value	0.052	0.438	0.038
	N	64	64	64
THADA_rs7578597	Effect size	-0.0550	0.016	-0.0125
	p-value	0.674	0.902	0.924
	N	61	61	61
ADCY5_rs11708067	Effect size	0.0278	-0.010	-0.1851
	p-value	0.828	0.938	0.146
	N	63	63	63
TCF2_rs757210	Effect size	-0.2421	-0.248	-0.2016
	p-value	0.072	0.065	0.136
	N	56	56	56
DGKB-TMEM195_rs2191349	Effect size	0.0683	0.047	0.1013
	p-value	0.634	0.744	0.479
	N	51	51	51
FADS1_rs174550	Effect size	0.0138	-0.168	-0.2057
	p-value	0.919	0.210	0.125
	N	57	57	57
IGF1_rs35767	Effect size	0.1291	0.118	0.1547
	p-value	0.309	0.354	0.222
	N	64	64	64
MADD_rs7944584	Effect size	-0.261	0.062	0.0086
	p-value	0.370	0.629	0.946
	N	64	64	64
CRY2_rs11605924	Effect size	-0.2313	-0.121	-0.0960
	p-value	0.086	0.374	0.482
	N	56	56	56
SLC2A2_rs11920090	Effect size	0.1216	0.077	0.0904
	p-value	0.346	0.551	0.485
	N	62	62	62
GLIS3_rs7034200	Effect size	0.0758	0.121	0.0959
	p-value	0.558	0.349	0.459
	N	62	62	62
PROX1_rs340874	Effect size	-0.0137	-0.033	-0.0147
	p-value	0.914	0.794	0.908

	N	64	64	64
C2CD4B_rs11071657	Effect size	0.0049	-0.038	-0.1413
	p-value	0.972	0.784	0.304
	N	55	55	55
VPS13C_rs17271305	Effect size	-0.0072	-0.053	-0.0533
	p-value	0.956	0.684	0.683
	N	61	61	61

Islet donors (n=64) were genotyped for 35 common single-nucleotide polymorphisms (SNPs) that have previously been associated with increased risk for T2D or glucose intolerance. Associations between donor genotype and the eigengene for the T2D-associated co-expression module, the eigengene for the 168 genes with islet-selective open chromatin (the T2D signature) and *SOX5* expression, respectively, was analyzed by linear regression using age, sex and BMI as covariates. Displayed p-values are not corrected for multiple testing.

Supplementary Table 13. Expression of 8 key markers of differentiated β-cells in human islets from non-diabetic (ND) and T2D donors

Gene symbol	Gene expression (a.u.) in ND (n=46)	Gene expression (a.u.) in T2D (n=19)	Ratio (T2D/ND)
GAD2	1105	891	0.81
GLP1R	320	208	0.65
MAFA	201	164	0.82
NKX6-1	261	191	0.73
PCSK1	3641	2557	0.70
PDX1	172	137	0.80
SLC2A2	221	113	0.51
SLC30A8	4421	3541	0.80

Gene expression as analyzed by microarray.
